# Independent organelle and organelle—organelle interactions: essential mechanisms for malignant gynecological cancer cell survival

**DOI:** 10.3389/fimmu.2024.1393852

**Published:** 2024-04-22

**Authors:** Ying Shen, Qiao-Chu Chen, Chen-Yu Li, Feng-Juan Han

**Affiliations:** ^1^ Department of Obstetrics and Gynecology, Heilongjiang University of Chinese Medicine, Harbin, China; ^2^ Department of Obstetrics and Gynecology, The First Affiliated Hospital of Heilongjiang University of Chinese Medicine, Harbin, China

**Keywords:** organelle, malignant gynecological cancer, mitochondria, endoplasmic reticulum, lysosome

## Abstract

Different eukaryotic cell organelles (e.g., mitochondria, endoplasmic reticulum, lysosome) are involved in various cancer processes, by dominating specific cellular activities. Organelles cooperate, such as through contact points, in complex biological activities that help the cell regulate energy metabolism, signal transduction, and membrane dynamics, which influence survival process. Herein, we review the current studies of mechanisms by which mitochondria, endoplasmic reticulum, and lysosome are related to the three major malignant gynecological cancers, and their possible therapeutic interventions and drug targets. We also discuss the similarities and differences of independent organelle and organelle–organelle interactions, and their applications to the respective gynecological cancers; mitochondrial dynamics and energy metabolism, endoplasmic reticulum dysfunction, lysosomal regulation and autophagy, organelle interactions, and organelle regulatory mechanisms of cell death play crucial roles in cancer tumorigenesis, progression, and response to therapy. Finally, we discuss the value of organelle research, its current problems, and its future directions.

## 1 Introduction

The cell is the basic unit of living activity; within eukaryotic cells, structural organelles with specific morphologies and functions carry out essential activities. Among them, mitochondria, endoplasmic reticulum (ER), lysosome, and others play key roles in the development of cancer-like diseases through their cellular activities.

Mitochondria are the main source of metabolic energy required for cellular function in aerobic eukaryotic cells, with roles in providing energy, regulating the cell cycle and programmed cell death, influencing metabolic processes including calcium signaling and the citric acid cycle, and often undergoing fission and fusion to help adapt their number and size to the metabolic activities of the cell (1). Mitochondria, the leading cancer-programming organelles, regulate nuclear function by controlling levels of various metabolites that affect gene expression. They are also critically involved in cancer development, proliferation, invasion, and metastasis, by altering the activity of cancer-related gene transcription and signaling pathways to impact mitochondrial dynamics, energy metabolism, mitochondrial apoptosis, and other cellular changes (2, 3). ER is a classical membrane organelle whose main functions include serving as a protein synthesis site for secreted and integrated membrane proteins as well as a subpopulation of cytoplasmic proteins involved in the synthesis of proteins and lipids, calcium regulation, and interactions with other organelles (4). Protein processing, modification, and folding in the ER are important regulatory processes that dominate cell function, fate, and survival; these can be mediated by changes in the response pathways to ER stress (ERS), which contributes to growth and metastasis in tumor-like diseases (5). The ERS response pathway is vulnerable to the influences of hypoxia, Ca^2+^ homeostasis, and other conditions under which ER proteins are misfolded and continuously accumulate, and which activates the unfolded protein response (UPR) (6). UPR activation is closely related to cancer cell survival regulation, angiogenesis, invasion, metastasis, and drug resistance (7). Lysosome is a key organelle that mediates active cellular processes like autophagy, apoptosis, necrosis, senescence, pyroptosis, and cancer cell ferroptosis (8). Lysosome cause cell mutations, degrade macromolecules (which provide nutrients for cancer cell proliferation), and produce hydrolase (which helps carcinogens destroy cell division regulation) (9). Therefore, the lysosome is also considered to be a regulator of homeostasis in cancer cells and organisms.

Although each organelle has specific, independent functions, they also coordinate to accomplish a series of important physiological functions; together, they constitute an organelle interaction network of fine labor divisions, collaborations, and close contacts (10). Almost all organelles can interact with one another, by forming membrane contacts, tight juxtaposition regions between different organelles, or different membrane types between intracellular membrane compartments (11, 12).

Mitochondrial autophagy, which interferes with the biogenesis of lysosome during mitochondrial dysfunction, is an important representative organelle interaction. Mitochondrial autophagy is a selective cellular autophagic process, in which mitochondria adapt to changes in their surroundings from external stimuli [e.g., nutrient deficiencies, reactive oxygen species (ROS) clustering, cellular senescence] which causes damage, triggers mitochondrial autophagy, and ultimately eliminate damaged mitochondria by targeted scavenging with lysosomes (13, 14). A specific cancer cell metabolic adaptation is the ability to apply specific autophagy forms, especially mitochondrial autophagy, which recycles intracellular components under conditions of metabolic stress or during anti-cancer therapy, affecting therapeutic resistance (15).

In addition, cell proliferation, invasion, metastasis, and other cellular activities occur in response to different stimuli. Thus, exploration based on different cell processes, which determine cell fate, is a better approach to addressing the urgent need for major medical treatments.

As the main site of ROS production during physiological cellular metabolism, mitochondria may be the main target of ROS-induced oxidative damage, and the close proximity of mtDNA to the respiratory chain as well as the absence of histone proteins and effective repair mechanisms have been suggested to be responsible for the greater susceptibility of mtDNA to oxidative damage than nuclear DNA (16). In the intraovarian environment, oocytes are the most abundant body cells in the mitochondria and rely primarily on these organelles for their ability to fertilize and early embryonic development, although they are highly susceptible to an oxidative environment that results in an imbalance in the levels of proteins required to maintain oocyte health and maturation (17, 18). Ovarian function is also closely controlled by mitochondrial protein homeostasis and mitophagy, and the initiation of the mitochondrial unfolded protein response (UPRmt) and mitochondrial autophagy maintains or restores ovarian and mitochondrial function (19). The changes in cervical tissue are often referred to as remodeling of the extracellular matrix, and the whole process is the result of a close relationship between biochemical and molecular pathways, tightly controlled by inflammatory and endocrine factors (20). Oxidative stress occurs when the production of ROS exceeds the antioxidant capacity; that is, the excess production of ROS overwhelms the antioxidant defense system and destroys lipids, proteins, and DNA, which leads to cell damage and cervical tissue dysfunction. For endometrial tissue, it has been shown (21) that excessive mitochondrial fission hinders endometrial stromal cells (ESCs) migration and induces apoptosis in ESCs. Storage and release of Ca^2+^ in oocyte maturation and fertilization are noteworthy features of the ER in female ovarian tissues, and appropriate calcium signaling responses can initiate oocyte development and embryogenesis, with the ER being central to initiating calcium signaling (22). For cervical tissues, it has been shown (23) that disrupting Ca^2+^ homeostasis and leading to ERS-associated cell death has a close impact on HeLa cell survival. Meanwhile, the UPR Ire-Xbp1 signaling pathway can be activated in metaphase cells (24). The lysosomal system, which degrades organelles and large protein aggregates that are impaired by endometrial growth, may be involved in the cyclic remodeling of endometrial cells by inducing apoptosis, which is most pronounced during the secretory phase, especially in the late secretory phase (25). The autophagy-lysosome pathway is an evolutionarily conserved process in eukaryotic cells that involves the transport of damaged organelles and proteins from the autophagosome to the lysosome, where they form autophagic lysosomes for degradation and are involved in the critical biological process of protecting primordial follicles that form the ovarian reserve (26). Autophagy-lysosome is closely related to HeLa cell viability (27). Thus, mitochondria, endoplasmic reticulum, and lysosomes all play different regulatory roles in the normal function of gynecological organs.

Mitochondrial autophagy, the classical mitochondrial-lysosomal organelle interaction mode, is a selective cellular autophagic process. Under the effects of external stimuli like nutrient deficiency, ROS clustering, and cellular senescence, mitochondria adapt to environmental changes, triggering quality impairment, autophagy, and ultimately eliminating damaged mitochondria via lysosome-targeted scavenging (13, 14). Also, the role of mitochondrial autophagy in cancer is complex and may influence cancer development through Parkin-PINK1-related signaling pathways and oxidative stress changes affecting cellular processes such as apoptosis, growth, proliferation, migration, and invasion. To ensure functional interactions between different organelles, organelles often communicate with each other through membrane contact sites. Among them, the mitochondria-ER interaction of mitochondria-associated ER membranes (MAM) is its representative mode, which plays an important role in Ca^2+^ exchange, mitochondrial dynamics, lipid metabolism, mitochondrial autophagy, and ERS. Ca^2+^ easily passes through the mitochondrial calcium unidirectional transporter (MCU) located in the ER and is later taken up by the mitochondria; and will promote the onset and development of different diseases represented by tumor-like diseases using related proteins, such as DRP1, PINK, GRP75, and Ero1α, as a starting point (28, 29). Therefore, organelle interactions have a special role in tumor diseases under normal conditions of action.

**Figure 1:**
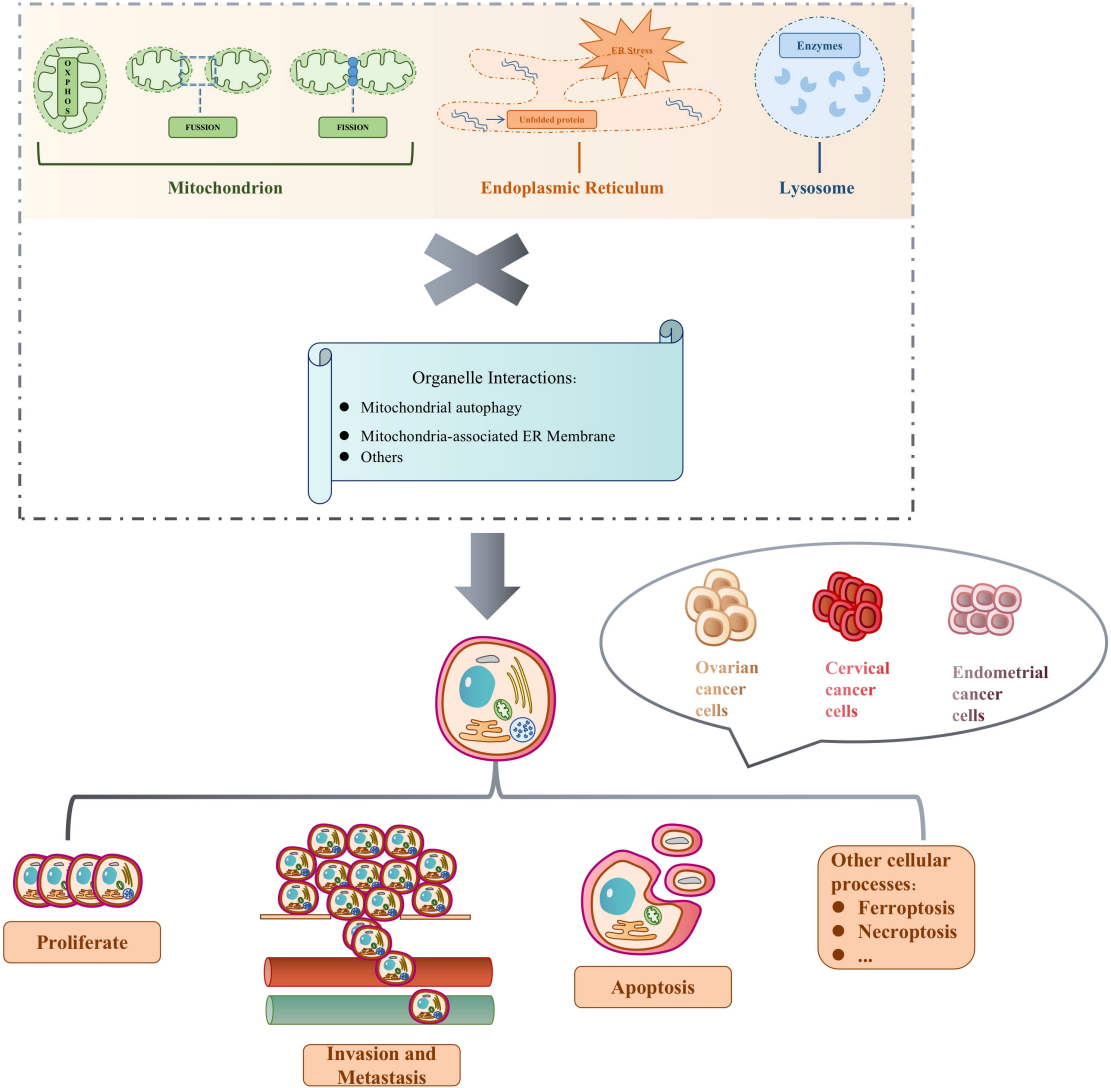
Independent organelles and organelle interactions determine cell fate in gynecological malignancies.

Ovarian, endometrial, and cervical cancers are considered the three most significant gynecological malignancies. Their incidences have increased in recent years, contributing to their status as significant silent global killers. Due to its hidden anatomical location and lack of clinical symptoms, ovarian cancer is mostly found in the advanced stage, often with metastasis and poor prognosis, with the 5-year survival rate of only 49%; cervical cancer and endometrial cancer have earlier clinical symptoms, with early stage in the majority of patients, and have a better prognosis, with the 5-year survival rate of more than 80%. However, once the tumor metastasis and recurrence occur, the prognosis will drop sharply, and the 5-year survival rate is less than 20%. The metastatic characteristics of the three clinically common gynecological malignancies are different: cervical cancer is dominated by direct spread; ovarian cancer is dominated by abdominal dissemination; and lymphatic metastasis of endometrial cancer is the most common clinical metastatic mode (30–32). Inhibiting gynecological malignancies is thus a major current medical challenge. Because mitochondria, ER, lysosome, and other independent organelles and organelle-organelle interactions are not only vital to the normal function of gynecological organs but also drive the development and fate of gynecological malignant cancer cells through direct participation in their energy metabolism (**Figure 1**), intervening in their signaling and guidance of cellular processes, promises to reduce the incidence of these cancers and lead to novel therapeutic interventions.

Mitochondria supply energy requirements mainly by their own oxidative phosphorylation reactions, while undergoing constant cell-based division and fusion. Under the influence of conditions like hypoxia and Ca^2+^ homeostasis, protein folding errors occur and accumulate, resulting in ER stress (ERS) and activation of unfolded protein response (UPR). Hydrolyzing enzymes in the lysosome constantly help regulate cell division. The three organelles work independently and in collaboration, triggering mitochondrial autophagy, mitochondria-associated ER membrane, and other organelle interactions to participate in a variety of biological processes to determine the fate of the three major gynecological malignancies, including ovarian, cervical, and endometrial cancers. Cell proliferation is the process by which a cell grows and divides into daughter cells in a rapid tissue growth mechanism. Apoptosis is the active cellular self-destruction action, generally including endogenous and exogenous apoptosis. Cell invasion is their ability to migrate from one area to another via the extracellular matrix, and is a normal response of both normal and cancer cells to chemical and mechanical stimuli. Metastasis begins when cancer cells leave the primary site and invade normal tissues or organs. Other cellular processes include ferroptosis and necrotic apoptosis, which are involved in guiding cell fate, along with cell proliferation, apoptosis, invasion, and metastasis. **Table 1** summarizes the abbreviation mentioned in the whole article.

**Table 1:** Acronym list.

Full English name	Abbreviation
Endoplasmic reticulum	ER
ER stress	ERS
Unfolded protein response	UPR
Reactive oxygen species	ROS
The mitochondrial unfolded protein response UPRmt	UPRmt
Endometrial stromal cells	ESCs
Mitochondria-associated ER membranes	MAM
Mitochondrial calcium unidirectional transporter	MCU
Extracellular signal regulated kinase1/2	ERK1/2
Epithelial-mesenchymal transition	EMT
Carnitine palmitoyltransferase 1A	CPT1A
Mitochondrial fission factor	MFF
Protein phosphatase 2A	PP2A
Protein kinase B	PKB/Akt
Oxidative phosphorylation	OXPHOS
Leukocyte-associated immunoglobulin-like receptor-1	LAIR-1
Peroxisome proliferator-activated receptor γ	PPARγ
Cancer-type organic anion transporting polypeptide 1B3	Ct-OATP1B3
Fatty acid β-oxidation	FAO
Peroxisome proliferator-activated receptor-gamma coactivator-1 alpha	PGC1α
Hexokinase 2	HK2
Mitochondrial permeability transition pore	MPTP
Voltage-dependent anion channel 1	VDAC1
Heat shock protein 70	HSP70
Spliced XBP1	sXBP1
Lysosomal protein transmembrane 5	LAPTM5
Epidermal growth factor	EGF
Epidermal growth factor receptor	EGFR
β-galactosidase	β-gal
Transcription factor EB	TFEB
Bcl-2/adenovirus E1B 19kDa interacting protein 3	BNIP3
Outer mitochondrial membrane	OMM
Light chain 3-II	LC3-II
5’ AMP-activated protein kinase	AMPK
Mitochondria-associated ER membrane	MAM
Leucine zipper/EF hand-containing transmembrane-1	LETM1
carboxy-terminal modulator protein	CTMP
Cytochrome c	cyt c
Apoptotic protease-activating factor-1	Apaf-1
Lysosome-associated membrane protein 3	LAMP 3
Uterine corpus endometrial carcinoma	UCEC
Inner mitochondrial membrane	IMM
Niclosamide	NIC
Interleukin-24	IL-24
Mitochondrial carrier 1	MTCH1
Mitochondrial-mediated ferroptosis	MMF
Benzyl isothiocyanate	BITC
Human papillomavirus	HPV
High-risk	HR
Histone deacetylase	HDAC
Maslinic acid	MA
Cathepsin D	CTSD
Cathepsin L	CTSL

## 2 Mechanisms of cellular organelles in ovarian cancer pathogenesis and therapeutic process

### 2.1 Mitochondria

#### 2.1.1 Ovarian cancer cell invasion

Sirtuin direct homolog SIRT6 can promote extracellular signal regulated kinase1/2 (ERK1/2), driving activation and phosphorylation of mitochondrial fission-associated protein DRP1, which positively regulates mitochondrial fission and reduces cell invasion by decreasing formation of stress fibers in ovarian cancer cells (33). Epithelial-mesenchymal transition (EMT) promoter Ets1 can promote EMT/invasion through DRP1-mediated mitochondrial fragmentation of ovarian cancer (34).

#### 2.1.2 Ovarian cancer cell proliferation

Carnitine palmitoyltransferase 1A (CPT1A) is characterized by regulating mitochondrial fission and function through the mitochondrial fission factor (MFF) succinylation mechanism to promote the proliferation of ovarian cancer cells (35). Protein phosphatase 2A (PP2A) is both a key negative regulatory molecule in cancers and an upstream molecule of protein kinase B (PKB/Akt), which is involved in cancer cell proliferation with the PP2A/AKT signaling axis (36). Major forms of cancer cell energy metabolism include aerobic glycolysis and oxidative phosphorylation (OXPHOS) pathways, by which they produce large amounts of ATP to meet the energy requirements for rapid proliferation. Leukocyte-associated immunoglobulin-like receptor-1 (LAIR-1), an inhibitory receptor containing immunoreceptor tyrosine inhibitory motifs expressed on most immune cells, is involved in macrophage formation by negatively regulating peroxisome proliferator-activated receptor γ (PPARγ), which regulates the promotion of both mitochondrial OXPHOS and glutamine metabolism to increase mitochondrial bioenergetic metabolism (37–40). Related experiments (41) have shown that under hypoxic conditions, expression of LAIR-1 in ovarian cancer HO8910 cells are significantly increased in a time- and dose-dependent manner, and that downregulation of LAIR-1 in HO8910 cells promotes cell proliferation and colony formation, and significantly increases ATP production, basal respiration, and maximal respiration; this also suggests that LAIR-1 is involved in mitochondrial bioenergetic metabolism regulation in ovarian cancer HO8910 cells.

#### 2.1.3 Ovarian cancer cell apoptosis

AKT, a serine/threonine kinase, is the direct upstream molecule of DRP1. It continuously regulates DRP1 activity and induces DRP1 phosphorylation based on Akt-DRP1 interaction, which is followed by mitochondrial division; thus, it is speculated that the PP2A/AKT/DRP1 signaling pathway is closely related to ovarian cancer cell mitochondrial division and apoptosis (42–45). This has been corroborated by relevant experiments (46).

#### 2.1.4 Ovarian cancer cell metastasis

Cancer-type organic anion transporting polypeptide 1B3 (Ct-OATP1B3), a SLCO1B3 family member, can directly interact with IGF2BP to trigger a series of changes that increase mitochondrial fatty acid β-oxidation (FAO) and OXPHOS activity, promoting metastasis of high-grade serous ovarian cancer (47).

#### 2.1.5 Ovarian cancer cell resistance

Drug-resistant ovarian cancer cells undergo a metabolic shift to OXPHOS, coordinated with mitochondrial network reorganization and component accumulation. Since peroxisome proliferator-activated receptor-gamma coactivator-1 alpha (PGC1α), a major mitochondrial biosynthesis regulator, is a key molecule in integrating and coordinating the transcriptional machinery of nuclear and mitochondrial DNA, it may be a target for improving chemotherapy efficacy and mediating ovarian cancer cell participation in cisplatin-resistant OXPHOS via nuclear-mitochondrial transcriptional feedback (48). Platinum-induced mitochondrial OXPHOS also contributes to ovarian cancer stem cell enrichment (49).

Mitochondrial apoptosis is a main mechanism in studies assessing cancer cell chemoresistance. Hexokinase 2 (HK2) defense protects mitochondria from apoptosis by maintaining mitochondrial permeability transition pore (MPTP) integrity and preventing apoptosis through mitochondrial expression and voltage-dependent anion channel 1 (VDAC1) binding (50). PGC1α can regulate apoptotic signaling by interacting with mitochondrial proteins; heat shock protein 70 (HSP70), an important member of the HSP family, transports proteins to mitochondria (51–54). Experiments have shown (54) that PGC1α knockdown decreases HSP70 levels and decreases HK2 and VDAC1 binding. Increased HK2 binding to VDAC1 and mitochondrial membrane potential after simultaneous use of a PGC1α inhibitor and overexpression of HSP70 suggests that PGC1α may affect HK2 and VDAC1 binding via HSP70 regulation. Thus, PGC1α may act on mitochondria to regulate the HSP70/HK2/VDAC1 signaling pathway and reduce apoptosis, promoting cisplatin resistance in ovarian cancer. This may be a new mechanism for cisplatin resistance in ovarian cancer, providing theoretical support for the clinical application of PGC1α inhibitors. It may also provide novel approaches for cancer screening biomarkers.

**Figure 2:**
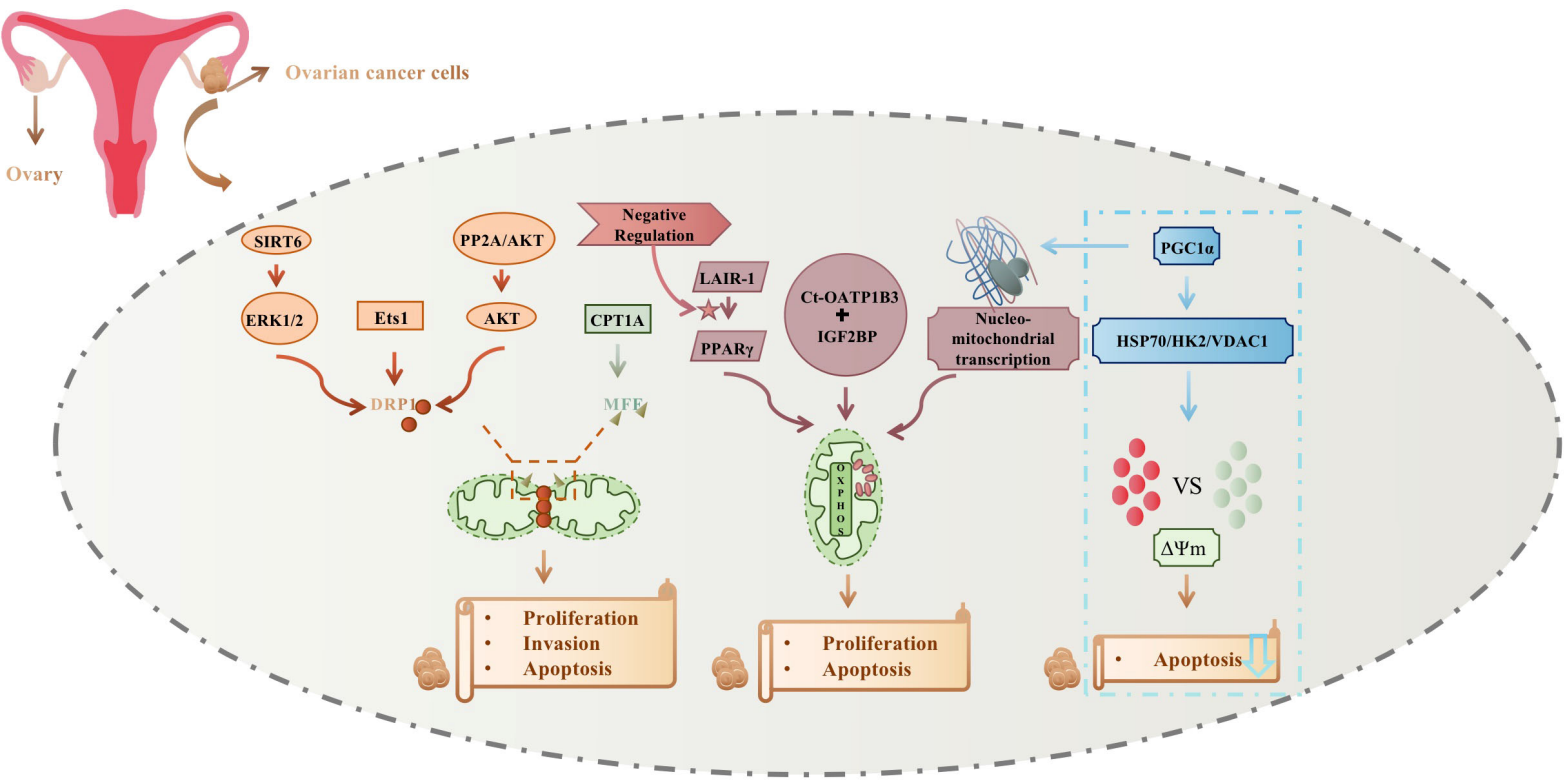
Mitochondrial relations with ovarian cancer development and treatment.

Mitochondria are mainly involved in the ovarian cancer cellular process by mitochondrial fission and auto-oxidative phosphorylation; changes also occur to nuclear-mitochondrial transcription and mitochondrial membrane potential, to control cell proliferation, invasion, and apoptosis. Mitochondrial fission regulation mainly affects DRP1 phosphorylation, which is mediated by ERK1/2, Ets1, and AKT, and MFF succinylation, which is mainly dependent on CPT1A. In contrast, LAIR-1 regulation of PPARγ, interaction of Ct-OATP1B3 with IGF2BP, and nuclear-mitochondrial transcriptional mediation of PGC1α affect mitochondrial OXPHOS activity. In addition, PGC1α promoted cisplatin resistance by modulating the HSP70/HK2/VDAC1 signaling pathway, affecting the binding of HK2 to VDAC1, causing alterations in mitochondrial membrane potential, and reducing the apoptosis of ovarian cancer cells. **Figure 2** summarizes mitochondrial relations with ovarian cancer development and treatment.

### 2.2 ER

ERS targets are involved in cancer cellular processes. Hypoxia, nutrient deficiencies, and a low pH tumor microenvironment can all cause accumulation of misfolded or unfolded ER proteins. This activates ERS and UPR, restoring cellular homeostasis or leading to cell death.

#### 2.2.1 GRP78 and UPR

Currently, the most important protein in this pathway is GRP78. With ERS, GRP78 expression increases and binds to the unfolded protein, causing dissociation of the three UPR sensors (i.e., IRE1, PERK, and ATF6), and initiating unfolded UPR protein responses through their respective signaling pathways (55). Elevated GRP78 levels in patients with ovarian cancer are associated with poor prognosis; they are also weakly expressed in cisplatin-sensitive ovarian cancer cells, mediating cisplatin-induced senescence (56).

#### 2.2.2 Ovarian cancer cell FOXK2 and stemness regulation

FOXK2, a stemness-specific transcription factor highly expressed in ovarian cancer, can bind to the intronic regulatory element of the target gene ERN1 to directly upregulate IRE1α expression; this leads to selective splicing of XBP1, after which spliced XBP1 (sXBP1) acts as a transcription factor to further promote other gene transcriptions. This suggests that the IRE1α/XBP1 axis promotes expressions of stemness-related genes, promoting ovarian cancer cell stemness, and that its regulation by FOXK2 is via the IRE1α/XBP1 axis (57).

#### 2.2.3 HERPUD1 and ovarian cancer cell apoptosis

HERPUD1, an important early ERS marker, also promotes ovarian cancer cell survival by maintaining autophagy and inhibiting apoptosis through the PI3K/AKT/mTOR and p38 MAPK signaling pathways (58). Non-apoptotic paraptosis is a novel mode of ovarian cancer cell death, the morphological features of which include ER and mitochondrial swelling and cytoplasmic vacuolization; initiated protein synthesis and ERS play important roles in this process, which also has a central role in ovarian cancer cells (56, 59, 60).

### 2.3 Lysosome

Lysosome, an organelle responsible for degrading cellular waste, recycling nutrients, and maintaining intracellular stability, is closely associated with ovarian cancer progression.

#### 2.3.1 Molecules associated with invasive ovarian cancer cell migration

E-cadherin expression downregulation and upregulated expressions of N-cadherin, vimentin and other EMT-related indexes result in cell polarity changes, reduced intercellular adhesion, loss of normal morphology, and enhanced migration and cellular invading abilities. Lysosomal protein transmembrane 5 (LAPTM5) can upregulate OVCAR3 expression, affecting EMT progression and altering cell migration and invasion (61).

#### 2.3.2 Specific enzyme actions

Abnormal glycosylation is a cancer cell characteristic, with a prevalent alteration being the enrichment of modified α2,6-linked sialylation of N-glycosylated proteins directed by ST6GAL1-sialyltransferase. ST6GAL1 is upregulated in many malignancies, including ovarian cancer, and its activity regulates epidermal growth factor (EGF) receptor (EGFR) transport dynamics following EGF-induced receptor activation via EGFR sialylation, which enhances cell surface post-activation receptor recirculation, while inhibiting lysosomal degradation to regulate ovarian cancer cells (62). β-galactosidase (β-gal), a hydrolyzing lysosome enzyme, serves as another important biomarker for cellular senescence and primary ovarian cancer (63).

#### 2.3.3 TRPML 1/TFEB calcium signaling pathway and ovarian cancer cell resistance

Transcription factor EB (TFEB), a member of the MiT/TFE family of transcription factors, is a major regulator of lysosomal biogenesis and autophagy, binding directly to lysosomal and autophagy gene promoters. Cisplatin induces TFEB nuclear translocation, upregulates downstream PD-L1 and PD-L2, and forms an immune-suppressive cancer microenvironment; this mediates cancer immune escape and drug resistance, and can be used to target TFEB inhibition to increase ovarian cancer cell cisplatin sensitivity (64). Lysosome can initiate calcium signaling through the TRPML 1/TFEB pathway, which promotes lysosomal cytotoxicity and clearance of accumulated material; lysosomal calcium channel inhibition attenuates lysosomal cytotoxicity and sensitizes drug-resistant ovarian cancer OVCAR8 cells to cisplatin treatment (65).

### 2.4 Organelle interactions

Though organelles have fine labor divisions, they also collaborate via close contacts to perform various biological processes under different cellular conditions. Dysfunctions in these interactions are closely related to the development of a variety of diseases.

#### 2.4.1 Interactions between mitochondria and lysosome

Mitochondrial autophagy is a well-described selective cellular process. Under the effects of external stimuli like nutrient deficiency, ROS clustering, and cellular senescence, mitochondria adapt to environmental changes, triggering quality impairment, autophagy, and ultimately eliminating damaged mitochondria via lysosome-targeted scavenging (11, 12). Mitochondrial autophagy is the classical mode of mitochondrial-lysosomal interactions. After detachment from the mother tumor, ovarian cancer cells are in a hypoxic environment; these cells initiate mitochondrial autophagy, which avoids hypoxic cellular damage and thus plays a key role in their generation of new metastases and establishment of blood supplies. Under specific conditions, Bcl-2/adenovirus E1B 19kDa interacting protein 3 (BNIP3) (an outer mitochondrial membrane [OMM] protein), interacts with the microtubule-associated protein 1 light chain 3-II (LC3-II) to mediate formation of autophagic vesicles and digestion of mitochondria (66). Experimental studies have shown (67) that expressions of BNIP3 and LC3-II, and mitochondrial autophagic activity, are significantly increased in ovarian cancer HO-8910PM cells under hypoxic conditions, thus initiating mitochondrial autophagy to maintain cell migration and invasion. BNIP3 expression inhibition significantly decreases LC3-II expression, suppressing mitochondrial autophagy in hypoxic environments; this results in typical hypoxic cell injury and inhibits cell migration and invasive functions. Thus, BNIP3 is hypothesized to be an important target in ovarian cancer metastasis. Depletion of the well-defined E3 ubiquitin ligase CRL4^CUL4A/DDB1^ upregulates phosphorylation of 5’ AMP-activated protein kinase (AMPK)α^Thr172^ and MFF^Ser172/Ser146^ to enhance mitochondrial fission, which in turn recruits DRP1 into mitochondria; its absence stimulates mitochondrial autophagy via the PINK1/parkin pathway, degrading dysfunctional and fragmented mitochondria and manipulating ovarian cancer cell chemoresistance (68).

#### 2.4.2 Interaction between mitochondria and ER

ER-associated interactions are also important to ovarian cancer cell survival. The Ca^2+^ regulatory network, centered on the ER, maintains intracellular Ca^2+^ homeostasis through coordinated interactions with mitochondria, lysosome, other organelles, and the plasma membrane. Ca^2+^ homeostasis dysregulation is closely related to malignant cancer occurrence and development. Increased cytoplasmic Ca^2+^ from the extracellular space or ER can trigger mitochondrial Ca^2+^ uptake, especially since the ER and mitochondria are physiologically and functionally interconnected at multiple sites. This MAM is involved in multiple processes, including Ca^2+^ homeostasis regulation, the main mechanism by which IP3R and VDAC promote mitochondrial Ca^2+^ uptake in the OMM (69). MAM formation, promoted by GRP75, is another potential target for overcoming ovarian cancer cisplatin resistance (70). The mitochondrial PHB2/OMA1/DELE1 pathway also coordinates during ERS to promote ovarian cancer responses to chemotherapeutic agents (71).

## 3 Cellular organelle mechanisms in endometrial cancer pathogenesis and therapeutic processes

### 3.1 Mitochondria

#### 3.1.1 Endometrial cancer cell migration and invasion

Expression of mitochondrial endometrial protein leucine zipper/EF hand-containing transmembrane-1 (LETM1) is higher in endometrial carcinoma tissues compared with atypical hyperplasia tissues, and higher in atypical hyperplasia tissues compared with normal tissues. Moreover, silencing LETM1 downregulates carboxy-terminal modulator protein (CTMP), reducing the activity, migration, and invasion, and inhibiting the malignant progression, of endometrial cancer (72). MCU upregulation enhances mitochondrial activity and promotes clone formation and migration of endometrial cancer cells; it also interacts with VDAC1 to enhance regulation of mitochondrial calcium uptake and promote endometrial cancer progression (73). Glucose metabolism in endometrial cancer cells is complex and mediated by glycolysis and mitochondria to ensure energy requirements. Factors affecting glucose metabolism may influence the initiation and progression of endometrial cancer and, unlike many other cancers that primarily exhibit increased glycolysis, it is more heavily OXPHOS-dependent. Endometrial cancer stem cells display higher mitochondrial membrane potentials, ROS, ATP levels, and rates of oxygen consumption, and their increased glucose uptake is associated with decreased lactic acid production and mitochondrial OXPHOS (74). Simultaneously, reduction of OXPHOS or complex I expression in different endometrial cancer types may contribute to less aggressiveness, in which the OXPHOS profile is associated with better disease outcomes, lower grading and staging, and endometrioid histology (75).

#### 3.1.2 Endometrial cancer cell apoptosis

Apoptosis is another major pathway for inducing effective cell death in cancer therapy. Its specific mechanism is mitochondrial regulation of the interactions among Bcl-2 family members, inhibiting Bcl-2 and activating Bax, which later induce formation of mitochondrial pores, leading to altered mitochondrial osmotic pressure and transmembrane potential loss, which releases mitochondrial membrane cytochrome c (cyt c) into the cytoplasm, where it binds to apoptotic protease-activating factor-1 (Apaf-1), forms apoptotic bodies, leads to caspase-9 precursor activation and subsequent cleavage with caspase-3 activation, degrading a range of cellular substrates and causing cell death (76–79). Release of high ROS levels in mitochondria and other initiators also stimulate the death receptor pathway, signaling different apoptotic systems and leading to apoptosis (80).

### 3.2 ER

#### 3.2.1 ER factor expression

Compared with normal endometrial tissues, positive expressions of derlin-1 and PAX2 proteins related to ER degradation are higher in endometrial cancer tissues. Moreover, on multivariate logistic regression analysis, International Federation of Gynecology and Obstetrics stages III–IV, differentiation degree medium-to-low, and lymph node metastasis were all independent risk factors for positive derlin-1 protein expression (81). ERS-induced ER peroxidase PRDX4 and the important molecular chaperone GRP78 are highly expressed in endometrial cancer tissues, indicating synergistic relations; their co-expression is an independent risk factor for endometrial cancer prognosis and overall survival (82).

#### 3.2.2 ERS

Metabolic disorders are a recognized risk factor for endometrial cancer. Impaired glucose metabolism accelerates dyslipidemia and promotes endometrial cancer progression due to ERS and hyperinsulinemia and saturated fatty acid-induced dyshomeostasis of Ca^2+^ and ERS (83). Risk profiles including TRIB3, CREB3L3, XBP1, and PPP1R15A are effective for predicting prognosis and immune relevance in patients with endometrial cancer (84).

### 3.3 Lysosome

#### 3.3.1 Lysosome factor expression

Like ER, lysosome-associated membrane protein 3 (LAMP 3) is expressed more highly in uterine corpus endometrial carcinoma (UCEC) compared with normal tissues. This is primarily regulated by a specific ceRNA network, the differential expression profile of which is closely associated with clinical and pathological features. Patients exhibiting high LAMP 3 expression tend to have shorter survival expectancies, making it a potent UCEC biomarker and potential diagnosis, treatment, and prognostic assessment candidate (85).

#### 3.3.2 Cancer factor regulation

Cancer changes in protein glycosylation are strongly associated with metastatic potential and immune evasion. One study comparing endometrial cancer and normal tissue samples observed 121 upregulated and 296 downregulated glycopeptides, with upregulation of approximately 80% of those involved in the lysosomal pathway (86). Genes associated with lysosomal biogenesis, including LAMP3, LAMP1, RIN3, and NPC2, are PAX8 targets, and regulating PAX8-DDX5 interactions promotes c-MYC-associated cell cycle progression in endometrial cancer in patients with the TP53 mutation (87).

### 3.4 Organelle interactions

Organelle interactions play an important role in endometrial carcinoma development and progression.

#### 3.4.1 Mitochondrial degradation with lysosome participation

Mitochondrial autophagy is a cytoprotective mechanism that allows cells to restore homeostasis by selectively removing damaged mitochondria, in response to lethal oxidative stress. A systematic study characterizing mitochondrial autophagy in endometrial cancer, to predict tumorigenesis and prognosis, showed that the endometrial cancer oncogene TOMM40 promotes cancer progression through mitochondrial autophagy-related pathways (88). KIF4A, in the kinesin protein superfamily, is closely related to mitochondrial autophagy; it interacts with TPX2, a protein involved in DNA damage repair in response to replication stress, to inhibit TPX2 ubiquitylation and enhance genomic stability in endometrial cancer cells (89).

In dysfunctional mitochondria, PINK1 is unable to import into the inner mitochondrial membrane (IMM) for cleavage and degradation, forcing it to stabilize on the damaged OMM (90, 91). Parkin, activated by PINK1 kinase activity, is an E3 ubiquitination ligase capable of labeling specific substrates for ubiquitination, thereby recruiting autophagosomes and encapsulating damaged mitochondria to complete mitochondrial degradation through fusion with lysosome (92, 93). In endometrial cancer, abnormal PINK1/parkin pathway expression promotes cancer occurrence and development. Mitochondrial autophagy process inhibition involved in the PINK1/parkin pathway induces apoptosis and inhibits cancer cell growth and proliferation. The mitochondrial autophagy gene E2F1 is also highly expressed in endometrial cancer, and its expression level is closely associated with poor prognosis in patients with UCEC or TP53 mutations (94).

The X chromosome protein-coding gene TIMM8A affects mitochondrial autophagy to influence immune infiltration and prognosis in UCEC (95). Enhanced chemodynamic therapy mediated by cancer-specific catalysts synergize with mitochondrial autophagy inhibition to improve endometrial cancer treatment efficacy (96). Estrogen regulation of intracellular calcium homeostasis in endometrial cancer cells originates from extracellular calcium endocytosis, rather than ER release, to regulate lysosomal activity and mitochondrial ROS, promoting endometrial cancer progression (97).

#### 3.4.2 ER and mitochondria coordination

INF2 protein in the ER can trigger mitochondrial division by recruiting DRP1 protein, and elevated INF2 has been significantly, negatively correlated with the hypocancerous FBXO7 protein in endometrial cancer specimens (98). Under energetic stress conditions, AMPK induces phosphorylation of INF2 (Ser1077), leading to its increased localization to the ER, which enhances DRP recruitment to mitochondria and promotes endometrial cancer cell growth (99).

**Figure 3:**
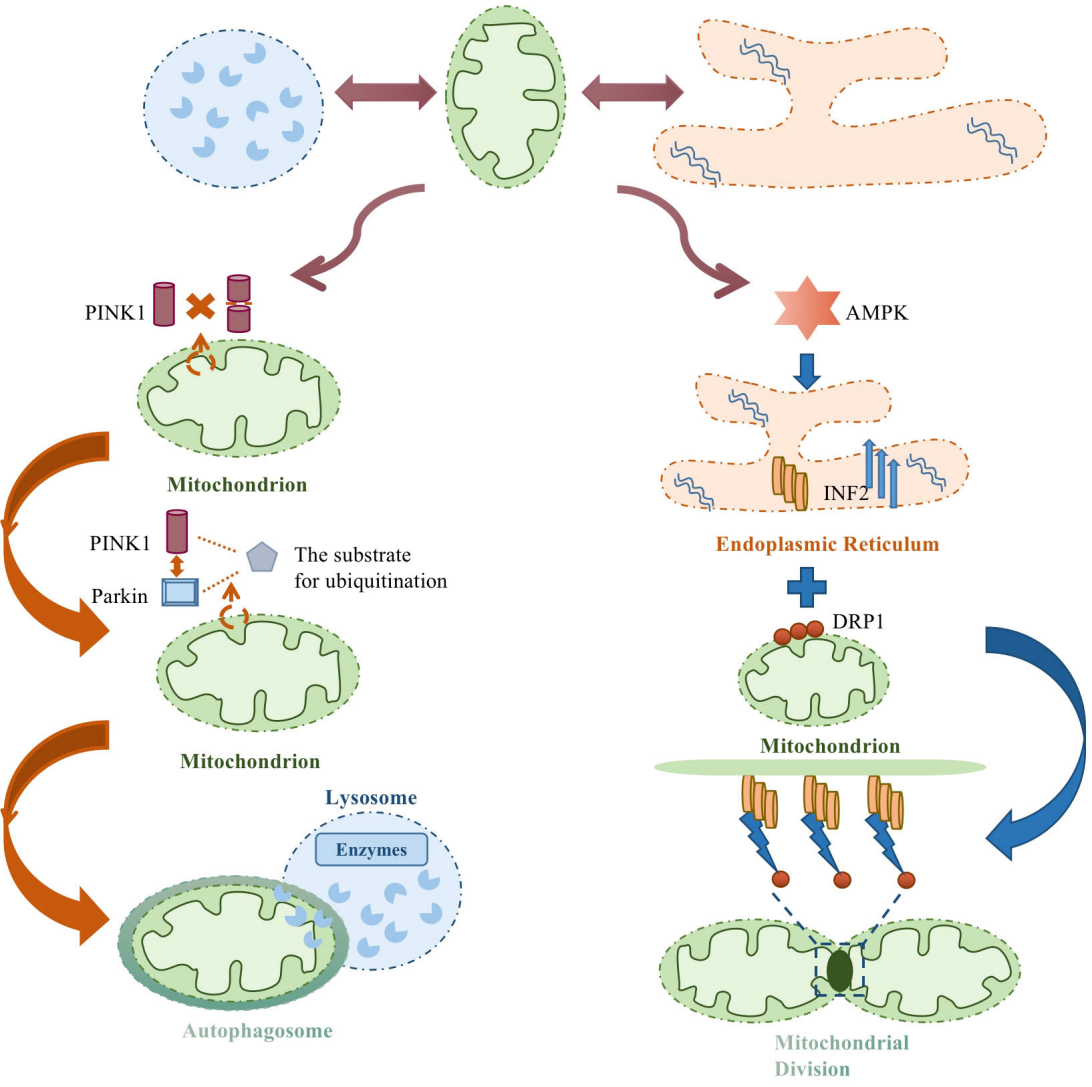
Endometrial cancer and organelle interactions.

Mitochondria-lysosome and mitochondria-endoplasmic reticulum are the main organelles involved in endometrial cancer cell progression. The former is mainly because PINK1 cannot be imported into the mitochondrial inner membrane for cleavage and degradation, and is forced to stabilize in the damaged mitochondrial outer membrane. Parkin activated by PINK1 kinase activity can label specific substrates for ubiquitination, thereby trapping the autophagosome, encasing the damaged mitochondria, fusing with the lysosome, and promoting mitochondrial degradation through hydrolase, completing mitochondrial autophagy. The latter, based on AMPK stimulation, increases INF2 localization in the ER and helps drive Drp1 loop assembly at mitochondrial contraction sites, triggering mitochondrial division. **Figure 3** summarizes endometrial cancer and organelle interactions.

## 4 Mechanisms of organelle involvement in cervical cancer pathogenesis and therapeutic processes

### 4.1 Mitochondria

Overexpression of FIS1 in HeLa cells inhibits cell proliferation and migration, promotes mitochondrial fission, leading to intracellular oxidative stress damage, and reduces cellular proliferation and migration functions (100). Mitochondrial carrier 1 (MTCH1) is a central mediator of mitochondrial-mediated ferroptosis (MMF) in cervical cancer, and its deletion disrupts mitochondrial OXPHOS and initiates FoxO1-GPX4 axis-mediated retrograde signaling, which increases ROS and ultimately triggers ferroptosis (101). NF-κB induces expressions of multiple cytokines and chemokines, stimulates transcription of proliferation-regulated genes, maintains mitochondrial homeostasis, and controls mitochondrial metabolic reprogramming to promote tumorigenesis; knockdown of IFI16, the main NF-κB-activating molecule, inhibits cisplatin-induced NF-κB (p65) entry into the nucleus and transcription of its target genes (IL-6 and cyclin D1), decreases mitochondrial membrane potential, exacerbates cisplatin-induced mitochondrial dysfunction, and increases HeLa cell sensitivity to cisplatin (102).

### 4.2 ER

Benzyl isothiocyanate (BITC) blocks the U14 cell cycle and has a role in upregulating ERS IRE1α gene, Atf4 gene, CHOP gene, and protein expression; this suggests that ERS plays an important role in BITC-induced cervical cancer cell apoptosis (103). The encoding gene CALCOCO1 binds to ATG8 family proteins, anchors to the ER and Golgi, participates in ER and Golgi autophagy, reverses the inhibition of autophagy after human papillomavirus (HPV) cell integration, and is mTOR pathway regulation-dependent (104). *In vitro* and *in vivo* model studies have shown that GRP78, a multifunctional calcium-binding ER protein, interacts with EIF3D to inhibit GRP78, and reducing EIF3D influence on the Warburg effect (i.e., aerobic glycolysis) and cell growth in an *in vitro* cervical cancer model (105). Knockdown of the ER membrane protein INSIG2 inhibits cervical cancer cell proliferation, migration, and invasion, while downregulating EMT-related gene expression levels (106).

### 4.3 Lysosome

Lysosomal membrane damage releases apoptosis-causing hydrolytic enzymes (e.g., histone proteases) (107). High-risk (HR) HPV E6/E7 (viral oncoprotein E6/E7) can inhibit autophagy by suppressing autophagic lysosome formation, and inhibition of autophagy diminishes cervical cell HR-HPV clearance, resulting in persistent infection and promoting cervical carcinogenesis (108). By activating the ROS/JNK pathway, promoting Bcl-2 phosphorylation, leading to the dissociation of Bcl-2 from BECN1, and inducing autophagy, enhanced autophagy lysosome formation can significantly downregulate cervical cancer cell HPV E6 and E7 expression levels and effectively inhibit their growth (107). Another study showed that after matrine was added, many autophagic lysosomes could be seen in HeLa and SiHA cells, and cell proliferation was affected, further confirming that cervical cancer cell proliferation may be affected by enhancing autophagic lysosome formation (109).

## 5 Discussion

### 5.1 Organelles and cell survival processes

While organelles have unique independent functions, maintenance of homeostasis and cellular function depends on their mutual contact and material transports. Independent organelle–organelle interactions have thus become a critical cell biology research focus.

#### 5.1.1 Organelles and key ovarian cancer cell survival processes

Mitochondria play an important role in ovarian cancer cells survival. Abnormal mitochondrial morphologic and functional changes are closely related to these cells’ characteristics of rapid growth, easy diffusion, and metastasis. Activation and phosphorylation of DRP1 and mitochondrial division-related proteins (e.g., MFF) regulate mitochondrial division processes and promote cell growth, proliferation, invasion, and apoptosis (33–35, 46). The SP70/HK2/VDAC1 signaling pathway is a core mechanism in MPTP openings, affecting apoptosis (54). LAIR-1 can participate in ovarian cancer cell mitochondrial bioenergy metabolism and OXPHOS, to promote cell proliferation (41). UPR activation regulates ovarian cancer processes, including cell cycling and chemotherapy resistance. Small molecules specifically targeting UPR signaling network components are expected to become potential ovarian cancer therapy interventions (110). The UPR marker HERPUD1 inhibits apoptosis through the PI3K/AKT/mTOR and p38 MAPK signaling pathways (58). GRP78 in ovarian cancer tissues is associated with poor prognosis, while ERS initiates UPR (56). Expressions of some molecules in lysosomes affect EMT progression and change cell migration and invasion abilities; thus, it may be an important ovarian cancer biomarker (61–63). Lysosome can initiate calcium signaling through the TRPML-1/TFEB pathway and regulate ovarian cancer cell drug resistance (65). The mitochondrial autophagy-associated PINK1/parkin pathway, BNIP3 molecules, and mitochondrial Ca^2+^ absorption promotion in the MAM might all be used to manipulate ovarian cancer cell metastasis and drug resistance (68, 70, 71).

#### 5.1.2 Organelles and key endometrial cancer cell survival processes

Expression of mitochondria-related molecules and OXPHOS affect endometrial cancer cell migration, invasion, disease severity, and prognosis (72–75); regulation of mitochondria is thus a primary way to induce apoptosis in endometrial cancer treatment (76–80). Expressions of many lysosomal and ER biogenic genes and factors affect endometrial cancer prognosis, effectively predict immune associations, and can be used as biomarkers (81, 82, 84, 85, 87). Thus, the diversity of organelle genes and factors diversify potential endometrial cancer treatment approaches. Organelle interactions in endometrial cancer mainly reflect the mitochondrial autophagy process, thus regulating cellular apoptosis (88, 94).

#### 5.1.3 Organelles and key cervical cancer cell survival processes

The degree of mitochondrial fission affects HeLa cervical cancer cell proliferation and migration (100). Regulation of gene transcription and protein expression can change mitochondrial membrane permeability, inhibiting cervical cancer cell proliferation through the mitochondrial apoptosis pathway, inducing apoptosis, and increasing HeLa cell drug sensitivity (102). Transcriptional expression of ERS-related genes and pathway-related factors induce apoptosis via the ERS pathway (103, 108, 111). Cervical cancer treatment might also be initiated through other cell death processes, including controlling ferroptosis through the FoxO1-GPX4 axis and retrograde signal transduction, which can inhibit cell growth (101).

#### 5.1.4 Organelles and survival processes by the three primary malignant gynecological cancers

In studies of endometrial, ovarian, and cervical cancers, mitochondria are important, with a large portion of this literature addressing the impacts of mitochondria on gynecological tumors. Mitochondrial dynamics like division, energy metabolism (e.g., ATP synthesis via the OXPHOS process), and other cell biology changes influence cellular health. The ER mainly controls ERS that occurs through molecular chaperones and respective UPR in responses to stress inducers (e.g., hypoxia, nutritional deficiencies) involved in cellular processes like cancer cell survival, invasion, metastasis, and chemoresistance. Lysosome specifically plays a prominent role in degradation, thus participating in tumor cell glycosylation processes and autophagy-lysosome formation by regulating the EMT process and expressions of associated proteins, influencing cancer invasive-metastatic potential and immune evasion. Molecules (e.g., genes, proteins) specifically targeting these three organelles are associated with cancer course prediction, development stage, and prognosis. Organelle interactions, including mitochondrial autophagy and MAM, also plays a central role in gynecological cancer cell survival. Different organelles work both independently and are interconnected to sustain specific cellular processes. This cumulative evidence provides new strategies and research directions for developing novel targeted organelle-based molecular therapies, detection and diagnosis methods, and approaches to assessing therapeutic efficacy.

### 5.2 Organelles and treatment

The similarities and differences between independent organelles and organelle-organelle interactions, as well as their application in their respective gynecological malignancies, play a crucial role in the survival and progression of cancer cells. Therefore, based on the independent organelle/organelle interaction, it is very meaningful to study the treatment status of ovarian cancer, endometrial cancer, and cervical cancer from this frontier perspective.

#### 5.2.1 Ovarian cancer treatment

In ovarian cancer treatment, different drugs that target independent organelle functions or comprehensive organelle interactions can achieve good efficacy.

##### 5.2.1.1 Causing or inducing apoptosis in ovarian cancer cells

Polyphyllin VII-enhanced mitochondrial localization of DRP1 is mediated by increased PP2A activity and decreased AKT activity, while the PP2A inhibitor LB100 attenuates polyphyllin VII-induced mitochondrial division and cellular apoptosis. Thus, interference with the mitochondrial translocation of DRP1 through the PP2A/AKT pathway may explain the effects of polyphyllin VII in ovarian cancer treatment (46). Cryptotanshinone inhibits mitochondrial complex I activity, reducing the ratio of NAD^+^/NADH and inhibiting ATP production, inducing AMPK activation, and thus controlling the energy reduction process, inhibiting ovarian cancer cell growth, and inducing apoptosis (112). In addressing ovarian cancer cell apoptosis resistance, the newly synthesized novel rhein derivative 4a was found to cause ERS and induce parapoptosis in ovarian cancer cells; however, its key targets and apoptotic inhibitory effects require further investigation (113). The anti-ovarian cancer effects of pimaric acid are mediated by increased p-PERK, PERK, AT-4, CHOP, and IRE-1 levels and cytotoxicity, based on ERS (114). The WEE1 inhibitor AZD1775 promotes ERS, upregulates GRP78, and activates PERK via CHOP to promote apoptotic signaling, while IRE1α-induces sXBP1 and maintains cell survival by inhibiting apoptosis (115). The autophagy inhibitor elaiophylin triggers paraptosis and preferentially kills ovarian cancer drug-resistant cells by inducing MAPK hyperactivation, providing a rational therapeutic strategy in refractory ovarian cancer (116).

##### 5.2.1.2 Inhibiting ovarian cancer cell proliferation or migration

*In vitro* studies have shown that OXPHOS activity is higher in the SKOV3 cell line, a more aggressive endocrine-responsive ovarian cancer, than in the less aggressive endocrine-responsive ovarian cancer OVCAR3. Furthermore, xihuangwan may inhibit expressions of PGC1α and TFAM by elevating ARHI expression in SKOV3 and HEY cells, increasing their oxidative stress levels, causing mitochondrial OXPHOS dysfunction, and thus inhibiting cell proliferation (117). The antimicrobial drug bedaquiline can target mitochondria to inhibit cancer cell growth, survival, and migration by decreasing mitochondrial respiration and ATP, and significantly enhancing cisplatin efficacy; this suggests that ATP synthase may be a selective target for overcoming cisplatin resistance in ovarian cancer (118).

#### 5.2.2 Endometrial cancer treatment

Drug therapy for endometrial cancer-targeting organelles has become a cutting-edge research area.

##### 5.2.2.1 Mitochondria as drug target

Metformin, a standard diabetes mellitus type 2 treatment drug, has shown promising results in cancer therapy by inhibiting mitochondrial OXPHOS and regulating AMPK, which reduces cell growth and proliferation. The mitochondria-targeting agent salinomycin also inhibits cancer stem cell proliferation, migration, sphere formation, and tumorigenicity, and induces apoptosis, including in endometrial cancer stem cells (74). Metformin treatment also reduces endometrial cancer stem cells by significantly decreasing mitochondrial membrane potential, targeting MYC signaling and mitochondrial bioenergetics in stem-like cells of endometrial cancer origin (119). Dioscin can promote human endometrial cancer cell apoptosis through the mitochondrial pathway, including Bax expression upregulation, Bcl-2 expression downregulation, ROS level increase, caspase 9/3 (in caspase family of cysteine proteases) activation, mitochondrial membrane permeability reduction, and, ultimately, OMM rupture and apoptosis. Dioscin also effectively activates marker genes and proteins (i.e., Fas, TNF-R1, caspase 8) associated with the death receptor-mediated pathway, confirming dioscin involvement in both apoptotic pathways (120).

##### 5.2.2.2 ER as drug target

CP41, a novel curcumin analogue, activates the H3F3A/proteasome-MAPK signaling pathway and significantly induces ROS levels to activate ERS, leading to apoptosis (121).

##### 5.2.2.3 Lysosome as drug target

Niclosamide (NIC) has emerged as a promising human endometrial cancer treatment by inducing Bax colocalization with lysosome and inhibiting Cathepsin B maturation from pro-cathepsin B, which induces lysosomal membrane permeability and releases hydrolase enzymes from the lysosome to cytosol, ultimately leading to cell death (122). Isorhamnetin is a flavonoid that affects ERS by modulating intracellular ROS levels, activating the endogenous mitochondrial apoptotic and exogenous death receptor pathways and ultimately inducing apoptosis, while inhibiting cell metastasis (123).

##### 5.2.2.4 Gene therapy and nanocatalytic system therapy

In addition to drug therapy research, organelle-based gene and nanocatalytic system therapies are also cutting-edge. The antitumor activity of interleukin-24 (IL-24), a unique cytokine cancer suppressor gene in the IL-10 cytokine family, has been demonstrated in a variety of tumors. IL-24 increases expressions of Bax and cyt c, but decreases Bcl-2, caspase-9, and caspase-3 expressions. Overexpression of IL-24 inhibits cell proliferation, migration, and invasion in endometrial carcinoma, but increases cell apoptosis; thus, it inhibits endometrial cancer cell growth by inducing apoptosis through the mitochondrial internal signaling pathway (124). While IL-24 has a specific, lethal effect on cancer cells, it does not adversely affect normal cells or tissues (125, 126). Thus, gene therapy may provide a novel approach to endometrial cancer treatment. ROS play a dual role in redox regulation. Moderate ROS-induced autophagy enables cancer cells to escape oxidative damage, while excessive or persistent ROS leads to autophagy-based cell death (127). Therefore, it is essential to determine whether autophagy induced by treatment methods (e.g., chemotherapy, radiotherapy, nanocatalytic system therapy) exerts cytoprotective or cytotoxic effects. Whether autophagy enhances or weakens the therapeutic effect is also an important question. nMIL-100 (Fe) nanocatalyst is catalyzed by H_2_O_2_ to produce highly oxidizing ·OH. Due to the excessive toxicity of ·OH around normal mitochondria, depolarization of mitochondrial membrane potential is subsequently initiated, further inducing mitochondrial oxidative damage and eventually apoptosis. Combining nMIL-100 (Fe) catalyzed by H_2_O_2_ with inhibitors of mitochondrial autophagy (e.g., Mdivi-1) exerts synergistic anticancer effects, which may aid development of more effective cancer therapies (128). Based on synergistic therapeutic inhibition of mitochondrial autophagy and ROS-based therapy, it will be important to target development of, for example, the nanocatalytic system, which can adapt to both enhanced oxidative attack and decreased antioxidant defenses, and thus may have great clinical translation value for cancer therapy.

#### 5.2.3 Cervical cancer treatment

To date, few studies have described the mechanisms by which organelles interact in cervical cancer, and drug research remains the primary focus. However, targeting independent organelle functions or comprehensive organelle interactions have been assessed in cervical cancer drug therapy.

##### 5.2.3.1 Mechanisms of cervical cancer cell proliferation inhibition

The PINK1/parkin-mediated ubiquitin pathway is the core mitochondrial autophagy pathway. Phosphorylation of ubiquitin leads to parkin activation, while acetylation of ubiquitin inhibits the formation and extension of ubiquitin chains. Through this mechanism, histone deacetylase (HDAC) activates mitochondrial autophagy by mediating parkin acetylation, thereby inhibiting cervical cancer cell proliferation (129). OMA1, which is present in the IMM, mediates hydrolysis of OPA1, resulting in the loss of its IMM fusion ability. Also through this mechanism, maslinic acid (MA) inhibits HeLa cell proliferation by upregulating cellular OMA1 and downregulating OPA1 and Bcl-2 protein expression levels (130).

##### 5.2.3.2 Mechanisms of cervical cancer cell apoptosis inhibition

Elevated Bax/Bcl-2 ratio leads to mitochondrial dysfunction and further mitochondria-mediated intrinsic pathway apoptosis, primarily intervening with MPTP opening, permitting release of cyt c and other apoptotic effectors, which later bind Apaf-1 and caspase-9 into an apoptosome complex, inducing cell death. In contrast, eleutheroside B can significantly increase Bax expression, decrease Bcl-2 expression, and increase the Bax/Bcl-2 ratio to increase cytoplasmic cyt c and downregulate anti-apoptotic protein survival, implicating the mitochondrial apoptotic pathway in cervical cancer (131). Similarly, HeLa cells undergo mitochondria-mediated apoptosis, with breakdown of mitochondrial membrane potential, significantly increased cytoplasmic cyt c, downregulated Bcl-2 expression, elevated Bax expression, activated caspase-3/-9, and significantly increased intracellular ROS; in contrast, ivermectin (IVM) can induce HeLa cell apoptosis precisely through the mitochondrial pathway (132).

Quercetin induces transcriptional expression of ERS-related factors PERK, eIF2α, ATF4, and CHOP, while transcriptional expression of the apoptosis-related factors Bax, caspase-3, and cyt-c is also significantly upregulated, and transcriptional expression of the anti-apoptotic protein Bcl-2 is markedly inhibited; this cumulatively suggests that quercetin induces cervical cancer cell apoptosis via ERS (133). Isoquercitrin promotes expression of GRP78, CHOP, and caspase-12 proteins to mediate ERS, and to further activate the apoptotic signaling pathway and induce cervical cancer cell apoptosis (134). In cervical cancer HeLa cells, aloe-emodin can produce lysosomal cytotoxicity, damage lysosomal membranes, increase histone activity, and induce apoptosis. Concurrently, emodin can enhance HeLa cervical cancer cell lysosomal membrane permeability, decreasing cathepsin D (CTSD) and cathepsin L (CTSL) activities and increased cytoplasm concentrations and leakage of lysosomal hydrolases into the cytoplasm, resulting in activation of caspases and inducing caspase-dependent apoptosis (107).

##### 5.2.3.3 Mechanisms of mitochondrial apoptosis and autophagy

LvHemB1 has anticancer agent potential via its interactions with the mitochondrial protein VDAC1. It causes mitochondrial membrane potential loss, increases ROS expression, upregulates apoptotic proteins (i.e., caspase-9, caspase-3, Bax), and induces mitochondrial apoptosis (135). Tandem mass tag proteomics and network pharmacology (136) has shown that Hed may inhibit advanced cervical cancer cell mitochondrial autophagy by blocking the autophagy effector fraction through modulation of the target network, which is dominated by HIF-1α, Src, Akt, and Stat3. Tanshinone I significantly inhibits cervical cancer SiHa cell proliferation, migration, and invasive ability in drug concentration- and time-of-action-dependent manners, interacting with BNIP3/NIX through hydrogen bonding and significantly affecting these cells’ differentially expressed gene profiles and metabolic reprogramming; it also promotes expressions of mitochondrial autophagy-related proteins BNIP3, NIX, and optineurin, which promote conversion of LC3I to LC3II and inhibit expressions of NDP52 and P62 (137).

##### 5.2.3.4 Mechanism of apoptosis in mitochondria and ER interactions

In HeLa cells treated with saikosaponin-A, levels of GRP78, CHOP, and saspase-12 increase the Bax/Bcl-2 ratio, release cyt c into the cytoplasm, and upregulate HeLa cell caspase-3cleavage. These results suggest that saikosaponin-A triggers apoptosis through mitochondrial and ERS pathways (138). Dezocine inhibits Hela cell viability in a dose- and time-dependent manners, activates ERS by upregulating expressions of GRP78, IRE1, and p-JNK, and weakens dezocine-induced apoptosis when the ERS pathway is blocked (139).

**Figure 4:**
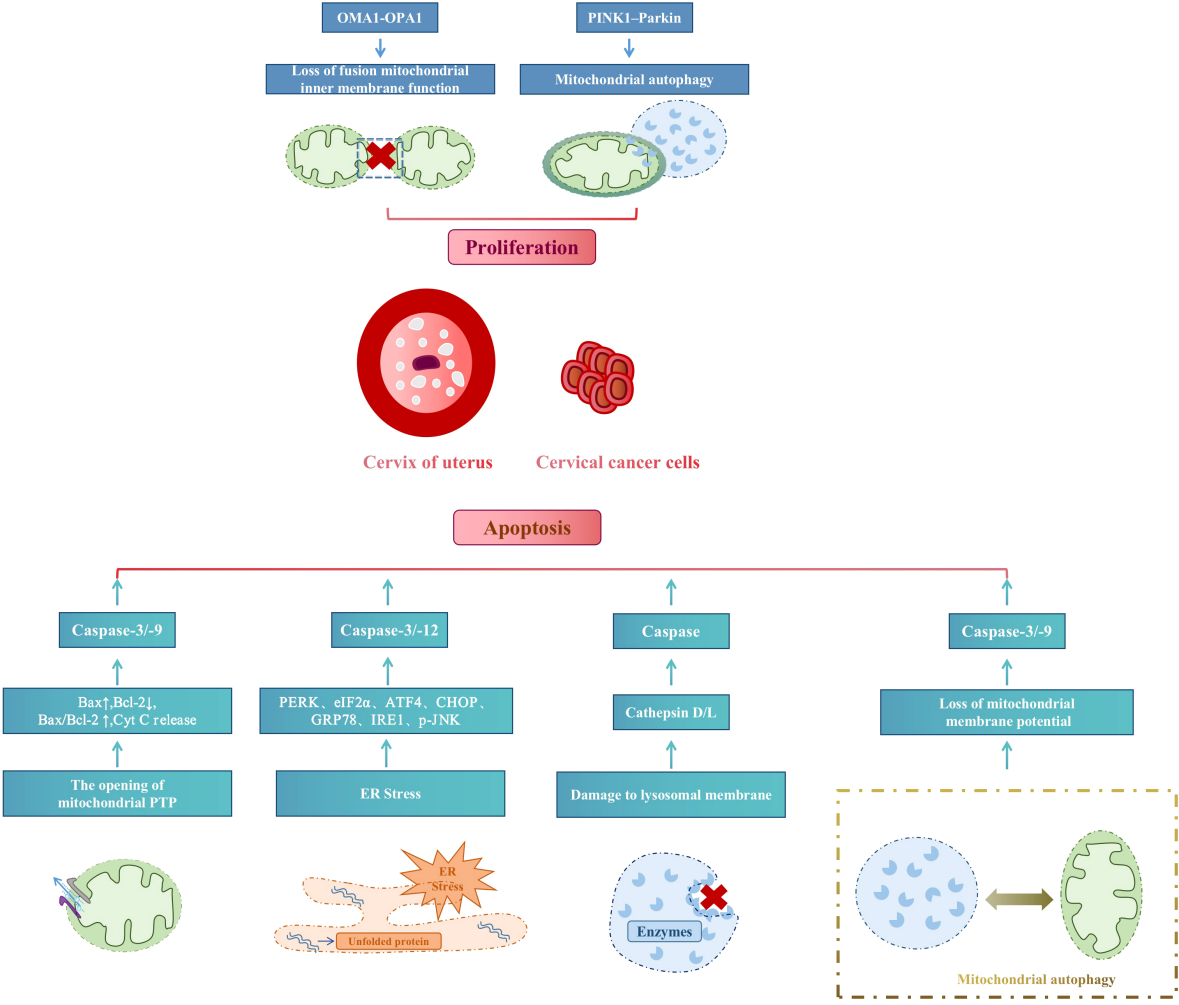
Mechanism of organelle action in cervical cancer treatment.

The core purpose of organelles in cervical cancer treatment is to reduce cell proliferation and increase cell apoptosis. PINK1/parkin mediated ubiquitin pathway-activated mitochondrial autophagy and OMA1 present in the inner mitochondrial membrane mediate inner membrane fusion protein OPA1 to control the mitochondrial fusion process and inhibit cell proliferation. In contrast, mitochondrial autophagy induces apoptosis through the opening of the mitochondrial permeability transition pore (MPTP/PTP), ERS, and lysosomal membrane damage. **Figure 4** summarizes the mechanism of organelle action in cervical cancer treatment.

**Figure 5:**
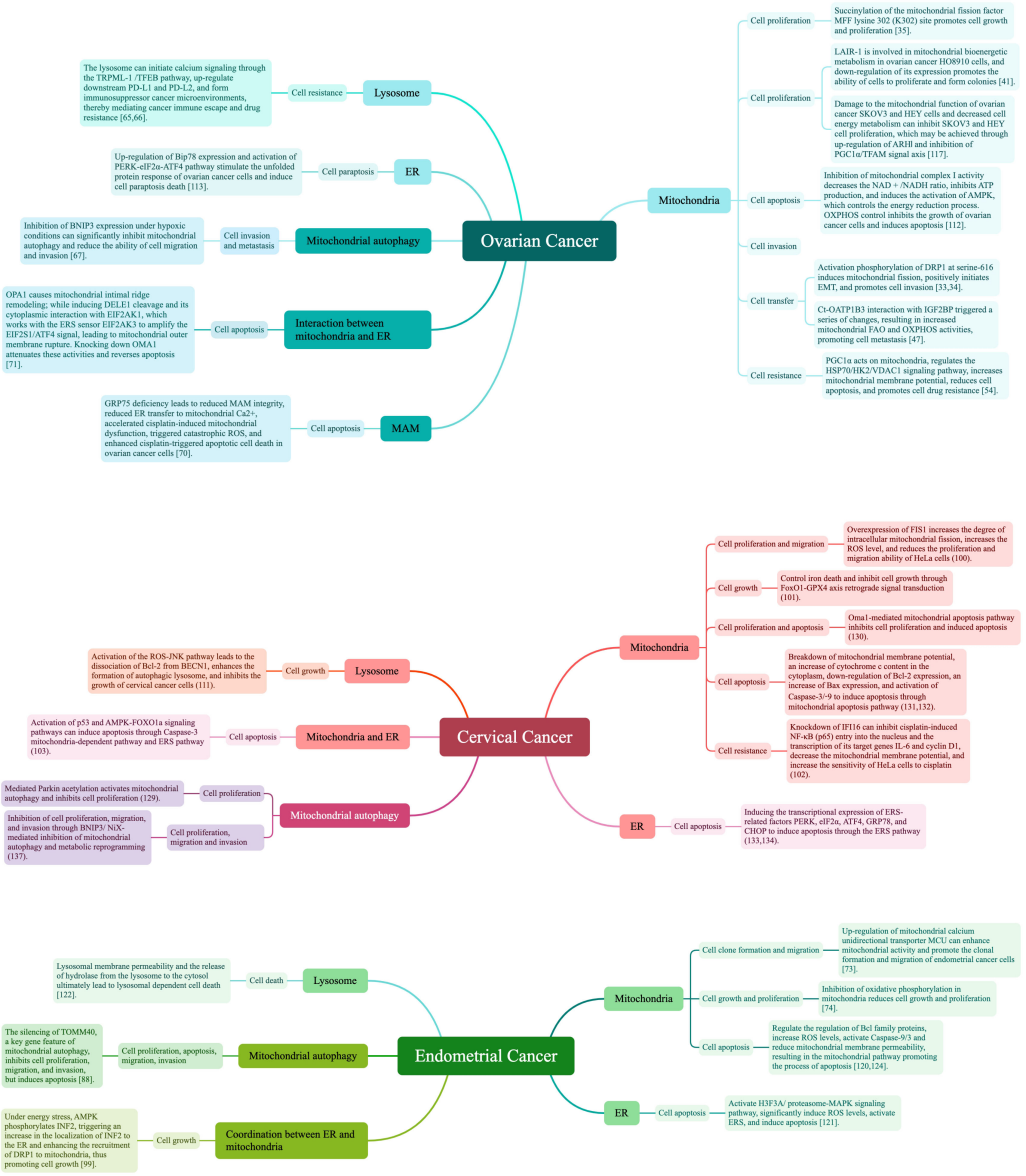
Literature summary of organelles and cell survival processes.

**Figure 6:**
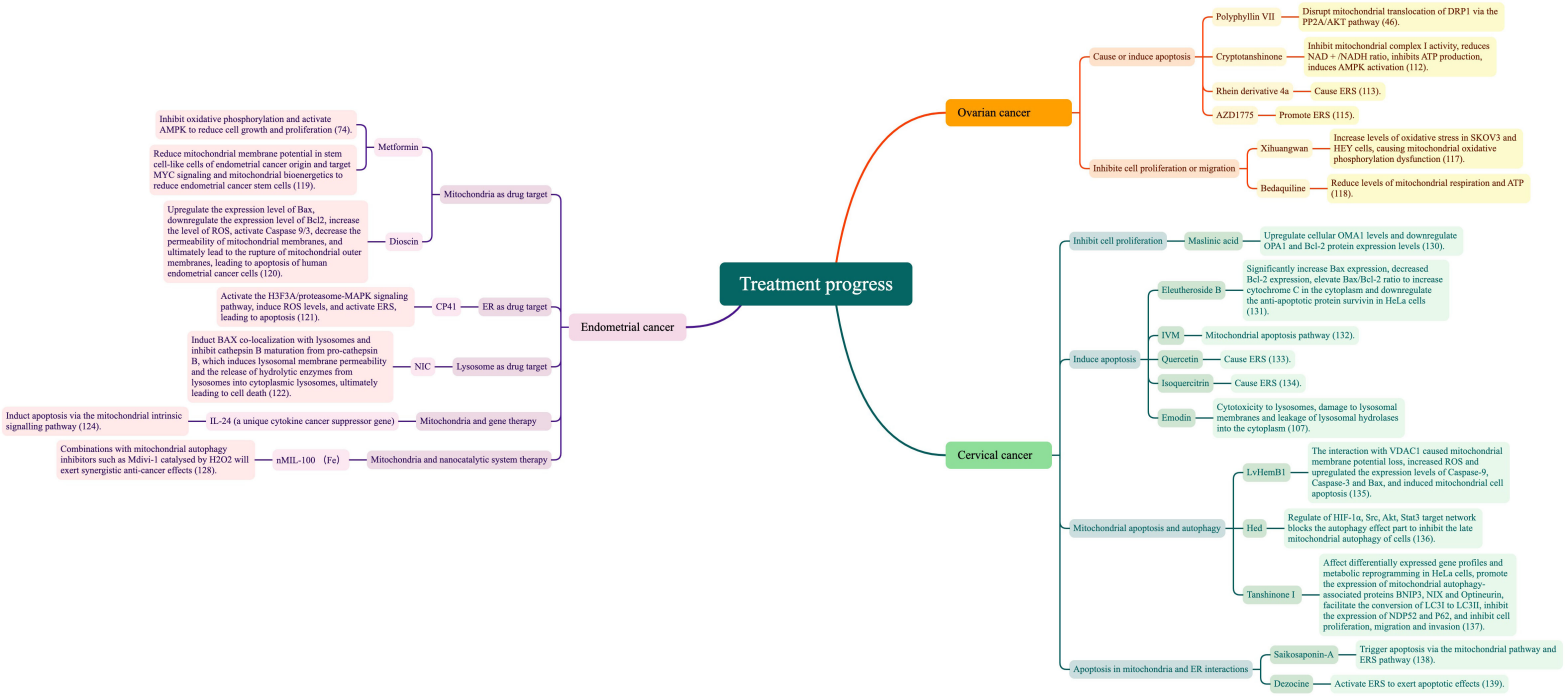
Literature summary of organelles and treatment.

To sum up, this review shows that development and survival of ovarian, endometrial, and cervical cancer cell processes are mediated through the mitochondria, ER, and lysosome, and through their mutual cooperation. **Figure 5** summarizes this current research literature. Also, we further explored the current status of the treatments that have been applied to ovarian, cervical, and endometrial cancers, and the available research literature is summarised in **Figure 6**.

Current organelle research in malignant gynecological cancers faces three primary problems. First, this research has mainly focused on certain specific organelles and organelle interaction modes, with the former dominated by mitochondria, and the latter relatively confined to mitochondrial autophagy. Second, research on the organelles and their interactions is still dominated by laboratory studies, while the efficacy of organelle-targeted molecular therapies, diagnostic accuracy, and the reliability of assessing therapeutic efficacy remain open questions in need of validation through clinical practices and applications. Finally, cutting-edge research into cellular processes is mainly centered on cell proliferation, invasion, and apoptosis, without involving multiple organelle types.

Herein, we content that four research directions remain to be further developed. First, expand the current research scope to explore the roles and mechanisms of other organelles (e.g., golgi, ribosome) and organelle interactions in gynecological malignancies. Second, highlight therapeutic targets and strategies, conduct practical efficacy analyses, and translate research results to clinical applications to improve gynecological tumor diagnostic and therapeutic qualities. Third, enrich the breadth of researching cell death regulation modes by other organelles (e.g., necrosis, ferroptosis) and other organelles that regulate cell death. Finally, based on the evidence in ovarian, endometrial, and cervical cancers, explore the specific biological characteristics, pathogenesis, and treatment options of other gynecological tumors from the perspective of organelles and their interactions.

## 6 Conclusions

Our main contribution herein is a summary of the links between key cell survival processes and the mitochondria, ER, and lysosome in the occurrence, proliferation, invasion, metastasis, and drug resistance contexts of the three major malignant gynecological cancers: ovarian, endometrial, and cervical. We also examined mitochondrial dynamics and energy metabolism, ER functional abnormalities, lysosome regulation and autophagy, ER–mitochondria and mitochondria–lysosome interactions, and the organelle regulatory mechanisms of cell death. This review revealed the similarities and differences among organelles and organelle–organelle interactions in respective gynecological tumor research and its applications. Current major organelle-targeting drugs and other therapeutics were also discussed.

We also describe the current dilemmas and difficulties within existing research and its applications, and propose issues and directions warranting further research: expanding the scope of organelle species research, determining the pathogenesis of different gynecological cancers within the organelle context, exploring new therapeutic targets and treatment strategies, and promoting clinical translation and research application.

Finally, although we have described herein the known importance of organelles, and their interactions, in the pathogenesis and treatment of gynecologic cancers, our knowledge of these roles remains limited. This currently restricts development of organelle-based therapeutic drugs and treatment approaches, emphasizing the need for further research that will both broaden and deepen our understanding.

## Author contributions

YS: Conceptualization, Funding acquisition, Writing – original draft, Writing – review & editing. Q-CC: Visualization, Writing – original draft, Writing – review & editing. C-YL: Writing – original draft, Writing – review & editing. F-JH: Conceptualization, Writing – review & editing.
